# Data on bone marrow stem cells delivery using porous polymer scaffold

**DOI:** 10.1016/j.dib.2015.12.014

**Published:** 2015-12-15

**Authors:** Ramasatyaveni Geesala, Nimai Bar, Neha R. Dhoke, Pratyay Basak, Amitava Das

**Affiliations:** a Centre for Chemical Biology, CSIR‐Indian Institute of Chemical Technology, Uppal Road, Hyderabad 500007, India; b Nanomaterials Laboratory, Division of Inorganic and Physical Chemistry, CSIR‐Indian Institute of Chemical Technology, Uppal Road, Hyderabad 500007, India; c Academy of Scientific & Innovative Research (AcSIR), 2 Rafi Marg, New Delhi 110001, India

## Abstract

Low bioavailability and/or survival at the injury site of transplanted stem cells necessitate its delivery using a biocompatible, biodegradable cell delivery vehicle. In this dataset, we report the application of a porous biocompatible, biodegradable polymer network that successfully delivers bone marrow stem cells (BMSCs) at the wound site of a murine excisional splint wound model. In this data article, we are providing the additional data of the reference article “Porous polymer scaffold for on-site delivery of stem cells – protects from oxidative stress and potentiates wound tissue repair” (Ramasatyaveni et al., 2016) [1]. This data consists of the characterization of bone marrow stem cells (BMSCs) showing the pluripotency and stem cell-specific surface markers. Image analysis of the cellular penetration into PEG–PU polymer network and the mechanism via enzymatic activation of MMP-2 and MMP-13 are reported. In addition, we provide a comparison of various routes of transplantation-mediated BMSCs engraftment in the murine model using bone marrow transplantation chimeras. Furthermore, we included in this dataset the engraftment of BMSCs expressing Sca-1^+^Lin^−^CD133^+^CD90.2^+^ in post-surgery day 10.

**Specifications Table**

**Table:** 

Subject area	Biology
More specific subject area	Stem cell delivery-biomaterials
Type of data	Text file, graph, figures and images
How data was acquired	DSC Q200 and TG Q50 TA Instruments, flow-cytometer Beckman Coulter MoFlo Legacy, microscope – confocal: Olympus FV1000; fluoroscence: Olympus U-RFL-T, thermal cycler Takara TP600, applied biosystems 7900HT real time PCR
Data format	Raw, analyzed
Experimental factors	Excisional splinting wound model, cell transplantation chimeras
Experimental features	Bone marrow stem cell (BMSCs) characterization, Scaffold-penetration by BMSCs-microscopic and zymographic analysis, BMSC transplantation chimeras (male cells into female mice)-qPCR analysis, BMSC engraftment at wound site and healing
Data source location	CSIR-Indian Institute of Chemical Technology, Hyderabad, India
Data accessibility	The data are with this article

**Value of the data**

• This data will be helpful for the research community that evaluates various biomaterials as cell delivery vehicles for tissue engineering and regenerative medicines.
• This data allows the scientific community to elucidate the penetration mechanism of cells into biomaterials.
• This data elucidates the site-specific homing and engraftment of transplanted stem cell chimeras using different routes of administration.

## 1 Data

**Fig. 1:**
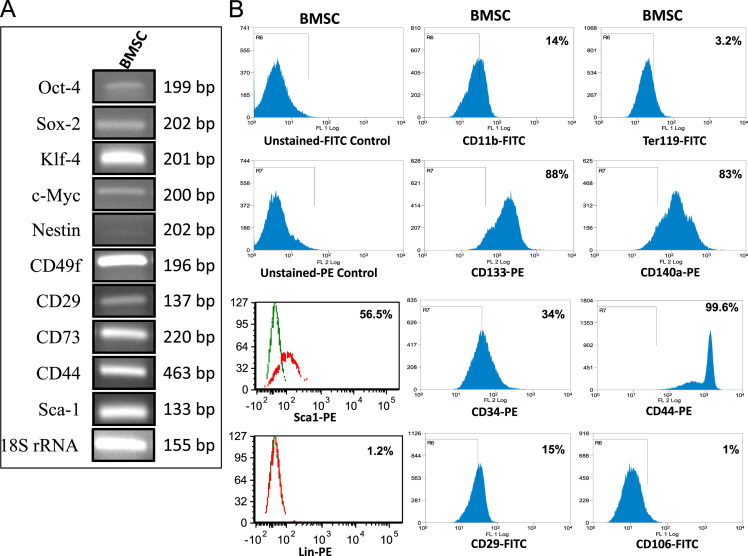
Characterization of mouse BMSC. (A) Expression of mouse gene-specific mRNA of pluripotency and stem cells marker genes in the isolated BMSC cell population as analyzed using semi-quantitaitve RT-PCR depicting Oct-4, Klf-4, Sox-2 and c-Myc along with nestin, CD49f, CD29, CD73, CD44 and Sca-1. 18 S rRNA expressions have been used in the same sample as an internal control. Results shown are representative images of experiments performed more than three times. (B) Differential expression of surface proteins such as Sca-1, CD11b, CD29, CD34, CD44, Ter119, CD106, CD133 and CD140a (Biolegend Inc, USA) and Lin (Miltenyi Biotec Asia, Singapore) on BMSC as analyzed using flow cytometry. Histogram on the extreme lefts of first and second rows along with green lined in the third and fourth row represents negative control (unstained FITC- or PE- control). The data reported are representative of three independent experiments each performed in duplicates.

**Fig. 2:**
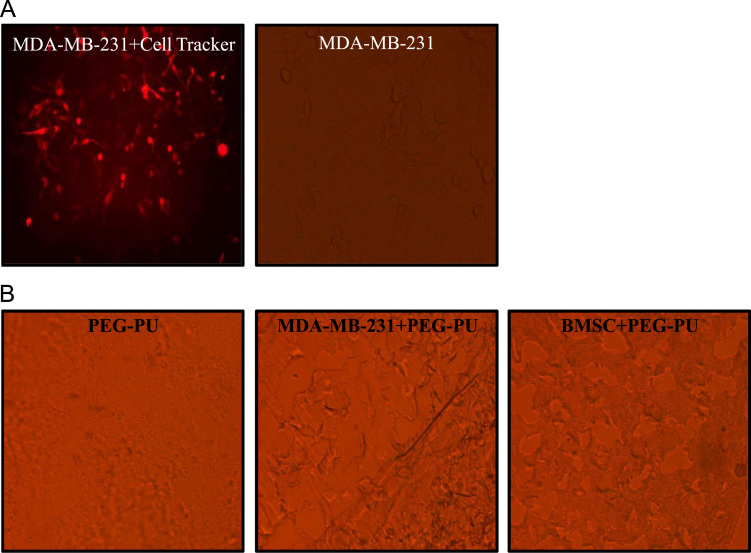
Cell penetrability in polymer network. (A) Representative images of MDA-MB-231 cells labeled with cell tracker dye (left) and phase contract image (right). (B) Representative phase contrast images of polymer network cultured with or without *MDA-MB-231* and *BMSCs*.

**Fig. 3:**
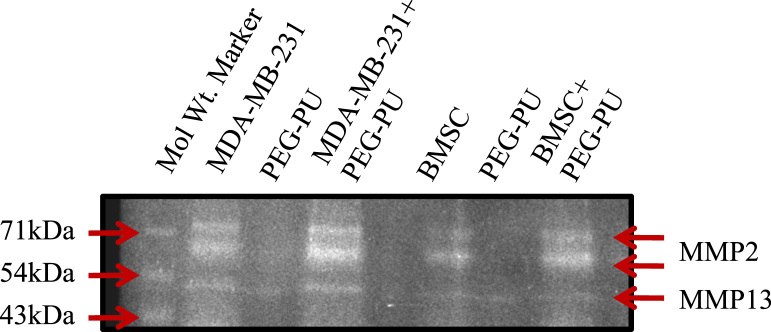
Collagen zymography indicating cell penetrability in polymer network. Representative zymogram images of MDA-MB-231 and BMSCs with or without polymer network.

**Fig. 4:**
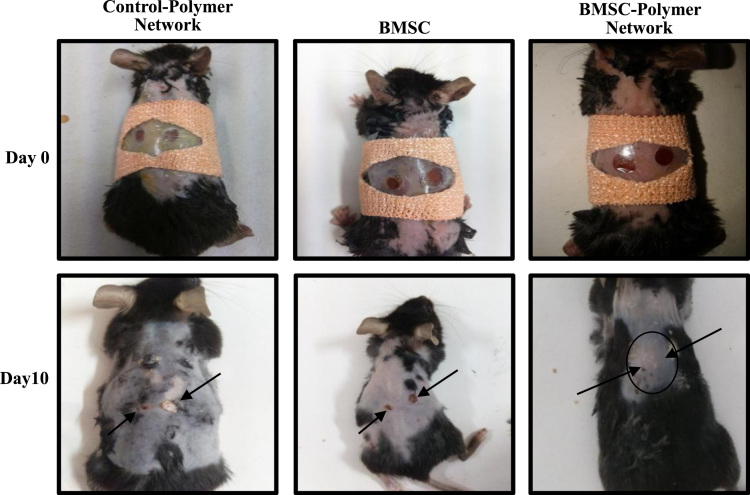
Bone marrow stem cells-polymer network mediated wound healing. Representative gross images of wound tissue healing at post-surgery day 0 (upper) and day 10 (lower, indicated with arrows) in mouse with vehicle control wound (left panel) or transplanted with either BMSCs (middle panel) or BMSCs-polymer network (right panel).

**Fig. 5:**
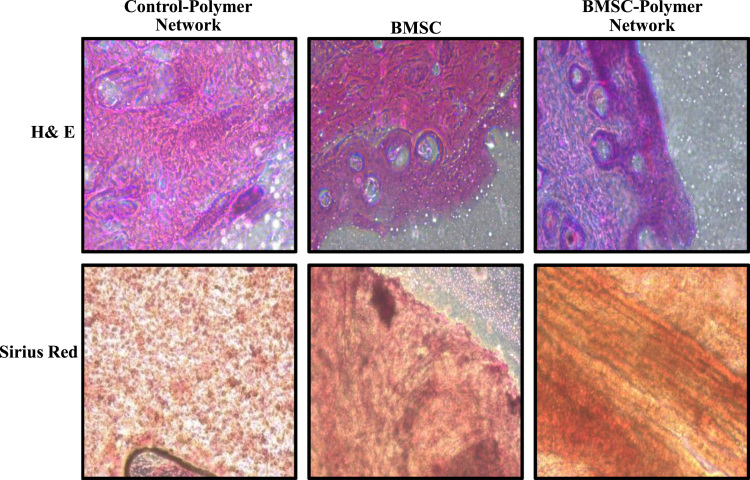
Histopathological analysis of wound tissue healing in presence of BMSC-polymer network. Representative photomicrographs of Hematoxylin–Eosin (upper panel) and sirius red (lower panel) stained tissue sections from vehicle control wound (left panel) or transplanted with BMSCs (middle panel) or BMSCs-polymer network (right panel) at post-surgery day 10 (*N*=5).

**Fig. 6:**
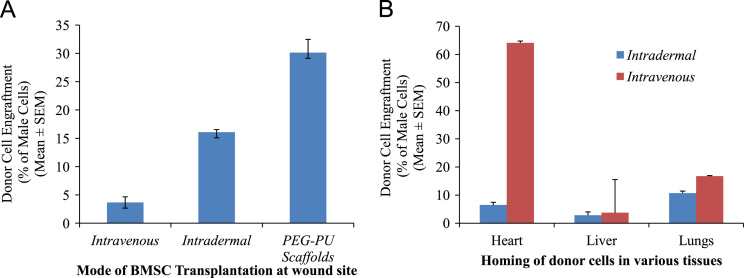
BMSC homing during transplantation. BMSCs of male *C57BL/6J* mice were transplanted in *C57BL/6J* female mouse wound injury model via different routes. (A) Percent of male cells at wound tissue when transplanted through various routes such as *intravenous, intradermal* and using PEG–PU scaffold. (B) Percent of male cells in various tissues such as heart, liver and lungs when transplanted through different routes.

**Fig. 7:**
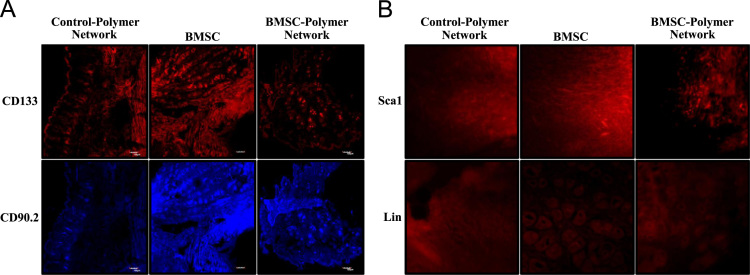
BMSC engraftment at the wound site. (A) Representative confocal images of regenerated wound tissue sections stained with BMSC markers, CD133 (upper panel) and CD90.2 (lower panel) at post-surgery day 10. (B) Representative immunofluorescence microscopy images of regenerated wound tissue sections stained with BMSC positive marker, Sca-1 (upper panel) and negative marker, Lin (lower panel) at post-surgery day 10.

We designed and synthesized a stable, biocompatible, pH-sensitive and enzymatically biodegradable castor oil-based porous polyethyleneglycol–polyurethane (PEG–PU) networks as scaffolds for delivery of BMSCs at an injury site that accelerates the wound tissue repair process (~50% faster) [1]. In this data, BMSCs isolated from murine bone marrow were characterized (Fig. 1A and B). Cells cultured in presence of polymer network were evaluated microscopically (Fig. 2) and biochemically for MMP-13 and MMP-2 activity (Fig. 3). Next, we utilized an *in vivo* syngenic murine excisional splinting wound model to evaluate the accelerated wound healing capacity of BMSCs when transplanted using polymer networks as scaffolds (Fig. 4). This dataset also depicts the histological analysis of wound tissue (Fig. 5). This data contains bone marrow transplantation chimeras (male cells in female mouse) for homing at wound site as well as different tissues (Fig. 6). Finally, immunostaining analyses of wound tissue sections to evaluate engraftment of transplanted BMSCs are included (Fig. 7).

## 2 Experimental design, materials and methods

### 2.1 Isolation and characterization of mouse BMSC

*C57BL/J6* mice were used for the isolation of BMSC. Briefly, bone marrow from tibias and femurs of 8 weeks old *C57BL/J6* mice were flushed out using αMEM. Subsequently, cells were plated using the same medium containing 10% FBS and 1% penicillin–streptomycin for 72 h with repeated changes of medium and subsequently passaged to perform experiments [2]. These cells were characterized using RT-PCR for pluripotency and stem cell markers Oct-4, Sox-2, Klf-4, c-Myc along with nestin, CD49f, CD29, CD73, CD44 and Sca-1 gene expressions. 18 S rRNA expressions have been used in the same sample as an internal control [3]. Flow cytometry analyses were also performed for various surface protein markers expression such as CD133, CD44, CD29, CD34, CD106, CD140a, Sca-1, CD11b, Ter119 and Lin. The above mentioned protocol was approved by the Institutional animal ethics committee (approval No. IICT/CB/AD/25/06/2014/13).

### 2.2 Cell penetration of polymer network assay

MDA-MB-231 (breast adenocarcinoma) cells and BMSCs were cultured in 24-well plates with a density of 1×10^5^ cells/well. Cells were labeled with deep red tracker dye (Molecular Probes, USA) as described in manufacturer׳s protocol. The cells were harvested and cultured in presence of PEG–PU for 24 h. The polymer networks were removed, washed and fixed with 4% paraformaldehyde. After embedding in sucrose, 30 µm sections were cut using cryotome [4]. Images were taken under fluorescence microscope (Olympus).

### 2.3 Zymography

MDA-MB-231 (breast adenocarcinoma) cells and BMSCs were cultured in presence and absence of PEG–PU for 24 h in a serum free medium at a cell density of 5×10^5^/well. Conditioned media was extracted and gelatin as well as collagen zymography was performed using the extracts at non reducing conditions as described earlier [5]. Gelatinolytic activity of MMPs was quantified using NIH ImageJ software.

### 2.4 Excisional wound splinting mouse model

8–10 weeks old *C57BL/6J* mice were used for generation of excision wound splinting model as described earlier [6]. Mice were anesthetized using an *intraperitoneal* (*ip.*) injection of sodium pento-barbital (50 mg/kg). The hair on dorsal side was removed by applying hair removal cream followed by disinfection of skin surface with povidine–iodine solution. Two symmetrical full-thickness excisional wounds were created besides the midline using 5 mm diameter sterile biopsy punch. Transplantation of BMSCs was performed by injecting *intradermally* (*id.;* 0.7×10^6^ cells) and on the wound surface (0.3×10^6^ cells). In a separate group, BMSCs were cultured in presence of 5 mm diameter polymer network and implanted as described above along with placing of the polymer network on the wound surface. A similar 5 mm diameter punched silicon splint ring was adhered around the wound and stitched at the corners to prevent the wound healing due to contraction of the skin and wound was dressed with transparent bandage. The above mentioned protocol was approved by the Institutional animal ethics committee (approval No. IICT/CB/AD/26/08/13/08).

### 2.5 Histopathology studies

Regenerated wound tissue samples from post-surgery day 7 and 10 of control wound, Vehicle control (PEG–PU), transplanted BMSC and BMSC-polymer network mice using 5 mm biopsy punch were fixed in 4% paraformaldehyde. The wounds were mounted on cryo-block using optimal cutting temperature (OCT) compound to make sections of thickness 10 µm using cryotome (Leica, Germany).

#### 2.5.1 Hematoxylin and eosin staining

Cross-sections of skin were mounted on slides and fixed using cold acetone for 10 min. After rehydration for 30 mins, sections were stained with hematoxylin for 5–15 mins and subsequently de-stained with 0.5% glacial acetic acid (if over stained) and washed. Sections were then incubated with eosin for 1–2 min followed by washing with 100% alcohol. The slides were washed with xylene and mounted for imaging under microscope [4].

#### 2.5.2 Sirius red staining

The sections were stained with Sirius red for 30 min to evaluate collagen deposition at the regenerated wound site. The stained sections were washed under running tap water for 2 min. The slides were counter stained with haemotoxylin, washed in xylene and mounted [7].

#### 2.5.3 Immuno-flouroscence analysis

The frozen sections were fixed with cold acetone and dried for 30 min. The slides were washed with PBS and incubated in normal goat serum (1:10 dilution) for 1 h. After blocking, the blocking buffer was drained and incubated with antibodies CD133-PE, CD90.2-APC, Sca-1-PE and Lin-PE for overnight at 4 °C. The slides were washed in PBST to remove unbound antibodies. The slides were subsequently washed in xylene and mounted using DPX mounting medium to view under confocal microscope (Olympus FluoView, Japan) [8].

### 2.6 Engraftment of BMSCs

BMSCs were isolated from male C57BL/6J mice and transplanted to female C57BL/6J mice through various routes of administrations such as *intradermal*, *intravenous* and/or along with the PEG–PU scaffolds at the wound site (*n*=5). BMSCs were cultured along with PEG–PU before implantation on wound as described earlier. 7 days post-surgery, wound tissue, along with other tissues/organs such as heart, liver and lungs were harvested from these mice followed by grinding in liquid nitrogen. DNA was isolated from the tissue using NucleoSpin tissue according to manufacturer׳s manual (Machery-Nagel, Germany). In a separate set of experiments to plot the standard curve, DNA was extracted from isolated BMSCs of male and female C57BL/6J mice, simultaneously. Real time PCR was performed for Y-linked zinc finger protein (Zfy-1) gene. Bcl-2 was used as reference control. Standard curve for the engraftment of male cells was plotted by increasing the % of male BMSC DNA with female BMSCs DNA as described earlier [9]. Zfy-1 gene expression level was calculated as the value of 2−*δ* Ct. Percent of male cells in various tissues of female mice was calculated using the standard plot [9].

### 2.7 Image analysis

The image files were opened using ImageJ software followed by conversion in 16-bit by various sequential steps provided in the software: Edit – Options – Scale. The staining was quantified by adjusting the threshold in the following steps: (I) “Image – Adjust – Threshold” – The auto/manual setting was used to select all the stained portions, (II) Process – to subtract background with rolling ball then – apply, (III) Process – binary – watershed, (IV) using “Analyze – Set Measurements” options finally selected the parameters to be measured. To make sure that only the selected gray level measurements are quantified, “Limit to Threshold” option was used, (V) “Analyze – Measure,” results appeared in a table form was saved and graphs were made by transferring this data to excel file, (VI) “Analyze – Analyze Particles” hava been used to measure individual feature profiles. Intensity measurements are performed within regions of interest by choosing the parameters at step V [10].
