# Supplementary material for: Data on bone marrow stem cells delivery using porous polymer scaffold
Source: Data Brief. 2015 Dec 15;6:221–8. doi: 10.1016/j.dib.2015.12.014 (PMC4707184; doi:10.1016/j.dib.2015.12.014)
Supplement: Supplementary file 2 — Supplementary material [file mmc2.docx]

*Differential Scanning Calorimetry and Thermogravimetry*

Thermal properties of polymers are important physical parameters that provide valuable insights into the overall behavior such as, miscibility, phase separation, segmental mobility, degree of crystallinity, thermal stability and degradation onset of synthesized matrices. Initial DSC studies revealed that the macromonomer, PEG (M_n_ ~ 4000) used for network synthesis and the component-II (PEGDME), both possess a very high degree of crystallinity as evident from the thermograms. A sharp endothermic melting peak (T_m_) at ~58 ºC for PEG with an enthalpy of ca. 192 Jg^-1^ corresponded to a predominantly crystalline bulk, with degree of crystallinity, %χ ~ 94 (see in Fig. 1C-a of the reference [1]). The glass transition temperature (T_g_) could not be ascertained owing to such a high degree of crystallinity. For the oligomer, PEGDME (M_n_ = 500) (see in Fig. 1C-b of the reference [1]), T_g_ was observed at ~-86 ^o^C along with a noticeably broad T_m_ at ~ 11 ºC and %χ~ 52, contributed by the significant amount of crystalline phase present. The broad endothermic peak (T_m_) witnessed with a prominent shoulder was due to polydispersity present in this low molecular weight oligomer. The glass transition temperature of the (50:50) semi-IPN matrix, ca. -71 ^o^C depicts a plasticized networked matrix. The Flory-Huggins interaction parameter i.e. miscibility of the components, PEG and PEGDME are similar and hence enhanced homogeneity of the synthesized matrix was expected. Consequently, a broad glass transition temperature with an inward shift signifying good miscibility of the two polymer components used was observed. Nevertheless, clear evidence for the presence of crystallized domains was indicated with the appearance of two endothermic peaks as indicated by T_m1_ and T_m2_. The broad lower melting temperature peak observed, T_m1_ ~ at 3 ^o^C was owing to the polydispersed oligomer PEGDME forming intra-molecular H-bonds. This observation suggests the existence of a small amount of exclusive crystalline PEGDME rich domains (microscopic phase separation) within the constrained confinements of the PEG-PU network. The appearance of the second endothermic peak (T_m2_ at ~ 35 ^o^C) was significantly shifted to lower temperature as compared to ~ 58 ^o^C for the pure PEG macromonomer. This was attributed to a mixed interface formed by entangled PEG of PEG-PU network and PEGDME chains that facilitates inter-molecular H-bonding. The T_g_ of porous polymer scaffold was observed at ~ -52 ^o^C, with clear indication of a completely amorphous polymer matrix in the absence of any melting region. This observation unambiguously indicates: *(i)* loss of inter-chain H-bonding owing to the absence of entangled PEGDME, *(ii)* PEG macromonomer are randomly entangled within the cross linked network, which in turn restricts formation of any intra-molecular H-bonding between the chains and hence cannot crystallize. The concurrent increase in glass transition temperature for the porous scaffold also suggests the loss of plasticization effect offered by PEGDME.

The thermal stability of the synthesized semi-IPN and porous PEG-PU network was further assessed employing thermogravimetry. An initial weight loss of ~ 1-3% was observed for both semi-IPNs and porous polymer scaffolds upto 150 ^o^C, possibly due to the loss of low molecular weight species, such as, absorbed moisture (see in Fig. 1D-a of the reference [1]). The differential plots clearly indicate a degradation onset temperature (T_0_) at and above ~210 ^o^C and ~ 240 ^o^C for the semi-IPN and polymer network, respectively (see in Fig. 1D-a of the reference [1]). Interestingly, the higher degradation onset for polymer networks devoid of component-II can definitely be attributed to the absence of low molecular weight PEGDME. T_0_ was followed by two stages of rapid weight loss within the temperature window of our study, as depicted in the figure (see in Fig. 1D-b of the reference [1]). The first stage, T_d1_ upto ~ 340 ^°^C was primarily assigned to cleavage and thermal destruction of ester bonds of castor oil. In the second stage, T_d2_ (~320 ^°^C-440 ^°^C), weight loss occurs presumably due to cleavage of urethane linkages of polymer network. The third stage of degradation beyond 450 ^°^C, T_d3_, was most likely due to advanced fragmentation of chain segments formed in the first and second stage of degradation. Encouragingly, the degradation onset indicates that polymers are appreciably stable, which is an important parameter to consider for their safe usage, handling and autoclaving.

- 1. *Synthesis and Physico-chemical Characterization of Polymer Networks*
     1. *Synthesis of Semi-Interpenetrating Polymer Networks (Semi-IPN)*: The process of preparing a typical semi-IPN matrix used in this study involves, reacting castor oil (-OH value ~ 2.7) with diphenylmethane-4,4'-diisocyanate (MDI) in requisite amounts for 1 h using THF as the solvent and nitrogen as inert atmosphere, forming a isocyanate terminated pre-polymer (stage-I). At the end of 1 h, this was charged with the polyether macromonomer (PEG, M_n_ ~ 4000) and N, N-dimethylaniline (DMA), room temperature catalyst to initiate the formation of a polyethylene glycol-polyurethane (PEG-PU) polymer networks, component-I (stage-II). Concurrently, the component-II, i.e. PEGDME (M_n_ ~ 500) having non-reactive end group in the preferred weight % was added within the system to intimately entangle at the growing polymer network. The reaction mixture was degassed and mixed vigorously for 30 minutes under inert atmosphere to obtain a uniformly homogeneous viscous mix of the desired composition. Finally, the viscous polymer solution was casted onto a teflon petri-dish, dried at room temperature for 24 h followed by curing at higher temperature and inert atmosphere to ensure the completion of isocyanate reaction (at 80 ^o^C for 48 h) forming a semi-IPN matrix. The free standing films so obtained have an average thickness in the range of ~ 0.08 - 0.12 cm. The semi-IPN samples used in the present study were synthesized with an equal composition of component-I and component-II (50:50; respective weight percentage) [2].
     2. *Realizing Porous Polymer Scaffolds:* The free standing films of the synthesized semi-IPN samples so obtained were wrapped in a Whatman filter paper bag and thereafter treated to a repeated soxhlet extraction process. Films were subjected to repeated swelling and drain cycles for 4-7 days against THF to extract out the PEGDME from the semi-IPN matrix completely, leaving behind a porous polymer network scaffold. The extraction was continued for another couple of days using deionized millipore water (18MΩ) to ensure an impurity free and sterile polymer matrix. Finally, the swelled porous polymer scaffolds were transferred into potassium phosphate buffer to carry out a series of bio-feasibility studies to demonstrate the viability and potential of these synthesized polymers.
     3. *Physico-Chemical Characterizations:* Fourier transform infrared spectroscopy was used to follow the formation of semi-IPN matrices in the mid-FTIR absorption range of 4000 - 400 cm^-1^ employing a Bruker ALPHA-T instrument. Typically, monomer/polymer samples (~2-5 mg) were grinded with KBr (~200 mg) and pressed into transparent pellets of approximate dimensions, Ø = 1.2 cm and t = 0.02 cm; followed by vacuum drying at 60 ˚C for 30 min prior to each run. The transmittance spectra collected for 256 scans with a resolution interval 2 cm^-1^, were corrected for baseline, atmospheric interference and also normalized when required before comparative evaluation. The polymer morphology was analyzed with scanning electron microscopy on a JEOL JSM-5600N. The cross-sections of the fractured semi-IPN matrices sputtered with gold and SEM images were acquired at different magnifications to ascertain the sample homogeneity, extent of phase separation and porosity. Differential scanning calorimetry was performed on a DSC Q200 differential scanning calorimeter (TA Instruments) under dry nitrogen atmosphere. Typically, a sample (5-10 mg) of polymer was loaded and hermetically sealed in an aluminum pan, rapidly cooled down to -150 ˚C using liquid nitrogen, equilibrated for 5 min and then heated up to 150 ˚C at scan rate of 10 ˚C min^-1^. The power and temperature scales were calibrated using pure indium and an empty aluminum pan was used as a reference. The analysis of thermograms was carried out using universal analysis software provided with the TA Instruments. The thermal stabilities of synthesized semi-IPNs were assessed by a TA Q500 modulated thermo gravimetric analyzer. 10 to 20 mg of semi-IPN samples were carefully weighed in an aluminum pan and TG scans were recorded at a ramp rate of 10˚C/min under inert atmosphere in the temperature range 35 to 600 ˚C.
  2. *Cell Culture:* Human Breast adenocarcinoma (MDA-MB-231; ATCC, USA) cell line was maintained in RPMI 1640 (Hyclone, USA) supplemented with 10% FBS (GibcoBRL, USA). Human Liver adenocarcinoma cell line (SK-HEP1; ScienCell, USA) was maintained in MEM (modified Eagle medium; Hyclone, USA) with 10% FBS. Primary cells isolated from mouse bone marrow were maintained in α-MEM (Sigma-Aldrich, USA) with 10% FBS. All the cells were maintained in sterile incubator with supply of 5% CO_2_ in a humidified atmosphere.
  3. *Trypan Blue Dye Exclusion – Viability Assay:* MDA-MB-231, SK-HEP1 and mouse BMSCs were plated (5 × 10^3^ cells/well) in a 96-well plate and cultured in presence or absence of polymer networks. Cell morphology was monitored microscopically after 24 and 48 h. Cells from different wells were trypsinized and counted for number and viability in a Neubauer counting chamber after staining with trypan blue dye, which selectively stains non-viable cells [3]. The results were expressed as relative cell number (compared with control samples normalized to 0%) with standard error of Mean from experiments performed thrice with three replicates.
  4. *Sulforhodamine B – Cytotoxicity Assay:* MDA-MB-231, SK-HEP1 and mouse BMSC were plated (5 × 10^3^ cells/well) in a 96-well plate and cultured with or without polymer networks. After 24 h, the cultured cells were fixed by means of protein precipitation using 20% TCA at 4 ºC for 1 h and subsequently washed for 5 times with RO water. After drying the plates for 24 h, SRB (0.05%) in 1% acetic acid solution was added to the wells and kept in dark for 30 min. Unbound SRB was removed and bound SRB was solubilized in 100 µl/well of 10 mM un-buffered tris base solution. The absorbance was read in a 96-well plate reader at 565 nm. Results indicate the cytotoxic effect of polymer networks on primary cells as well as cell lines [4].
  5. *MTT – Proliferation Assay:* Effect of polymeric networks on the proliferation of primary cells as well as cell lines was evaluated using MTT test. MTT (3–4, 5–dimethylthiazol–2–yl)–2, 5–diphenyltetrazolium bromide) test is based on the conversion of tetrazolium soluble salt into formazan which is mediated by mitochondrial NAD and NADH–dehydrogenases present in the viable cells. Briefly, the cells were seeded at a 5 × 10^3^ cells/0.1 ml density in 96-well plates and incubated for 48 h in presence or absence of polymer networks followed by addition of MTT reagent and further incubation for 4 h in the dark. The formazan crystals formed were then dissolved using DMSO and the end product was quantified using a microplate spectrophotometer (Perkin Elmer Enspire, Germany) at a wavelength of 570 nm [5]. The percent of viable cells cultured with the polymer networks was calculated with reference to the control sample (cells cultured without the polymer considered as having a viability of 100%).
  6. *Hoechst Staining – Apoptosis Assay:* BMSCs and MDA-MB-231 cells were cultured on cover slips with and without polymers in a 6-well plate as described above. The cells were fixed with 4% paraformaldehyde and incubated with Hoechst stain (2 mg/ml) for 30 min. The excess stain was washed with PBS and the cover slips were mounted on a slide for imaging under confocal and/or fluorescent microscope [6].
  7. *RNA Isolation, cDNA Synthesis and Quantitative RT-PCR (qPCR) Analysis:* MDA-MB-231 cells were cultured in presence and absence (control) of polymers for 24 h. Cells in presence of polymers were treated with p-akt inhibitor wortmannin (100 nM) and p-ERK inhibitor PD 98059 (30 µM) for 24h. After the incubation polymers were removed from medium, washed and homogenized to obtain cell lysate. RNA was extracted from control cells as well as cells penetrated inside the polymer using Ribozol according to manufacturer’s instructions. 1 μg of template RNA was utilized to synthesize cDNA using Verso cDNA synthesis kit (Thermo Scientific, USA). Further SyBR Green PCR method was used along with specific forward and reverse primers of MMP-2, MMP-7, MMP-9, MMP-13, TIMP-1, TIMP-2 in an ABI step-one plus instrument (ABI, USA). ACTB and GAPDH expressions were used in the same reactions of all samples as an internal control [7].
  8. *Western Blot Analysis:* MDA-MB-231 cells were cultured in presence of polymer networks for 48 h. Also polymer networks were separately, incubated with only medium. The polymer networks were removed from the wells and homogenized using RIPA lysis buffer containing protease inhibitor cocktail. Protein extracted from the cells penetrated inside the polymer was subjected to SDS-PAGE electrophoresis followed by immunoblot analysis using primary antibodies against phosphorylated Akt (p-Akt), total Akt, p-ERK and total Erk (Pierce Antibodies, USA) [7].
  9. *Exposure of H_2_O_2_:* MDA-MB-231, SK-HEP1 and mouse BMSCs were plated (5 × 10^3^ cells/well) in a 96-well plate and cultured in presence or absence of polymer networks. The cells were exposed to H_2_O_2_ at an increasing concentrations of 0.1, 1 and 10 µM. MTT assay [5] was performed as described above to evaluate the proliferative potential of cells whereas Hoechst staining [6] was performed to evaluate the apoptosis of cells in presence of polymer networks.
  10. *Gene expression studies:* RNA was extracted from wound tissue samples of control, vehicle control (PEG-PU), BMSC, BMSC+PEG-PU post surgery day 7. The forward and reverse primers of inflammatory cytokines (pro-inflammatory cytokines IL1, IL2, IL3, IL5, IL6, IL8, IL 17, IL18, IFNγ, TNF-α and anti-inflammatory cytokines IL10, IL13), anti-oxidant enzymes (Catalase, SOD1, SOD2, GPx1, GPx2) along with endothelial cell markers (VEGFR1, VEGFR2, VEGFR3, Nrp1, Nrp2, Tie2, α-SMA) was used to perform quantitative PCR analysis as described earlier [8].
  11. *DHE Staining:* Post-surgery day 7 samples from all the four groups; control, vehicle control, BMSC transplanted with and without polymer network wounds were embedded in 4% paraformaldehyde and sections were made with thickness of 10 µm using cryotome. The sections were stained with Dihydroethidium (DHE) (10 µM) solution for 30 min. DHE or hydroethidium is a compound which penetrates into the cells and interacts with O_2_^−^ thereby forms a byproduct known as oxyethidium. This product upon interaction with nucleic acids emits red color qualitatively detected by confocal microscope [9].
  12. *Biochemical Antioxidant Enzyme Assays:* The regenerated wound tissue samples harvested on post-surgery day 7, using 5 mm biopsy punch with same diameter of excision were homogenized in respective sample assay buffers for antioxidant enzyme analysis such as Catalase, SOD and GPx.
      1. *Catalase (CAT):* The catalase activity assay was performed by the method of Aebi (1984) [10]. The homogenized sample in phosphate buffer (pH 7.2) was centrifuged at 12000 rpm for 30 min. The supernatant collected was used for analyzing catalase activity in presence of H_2_O_2_. The decrease in the absorbance measured at 240 nm represents the rate of decomposition of H_2_O_2_ by the catalase present in the sample.
      2. *Superoxide Dismutase (SOD):* Both cytosolic (Cu/ZnSOD) and mitochondrial (MnSOD) were extracted from the samples using differential centrifugation technique as suggested by the manufacturer’s protocol, superoxide dismutase assay kit (Cayman chemicals, USA). In this assay, xanthine oxidase and hypoxanthine system generates the superoxide radicals. One unit of SOD defined as the amount of enzyme needed to exhibit 50% dismutation of the superoxide radical 11].
      3. *Glutathione Peroxidase (GPx):* GPx activity was assayed using GPx assay kit (Cayman Chemicals, USA). This assay measures GPx activity indirectly by a coupled reaction with glutathione reductase (GR). Oxidized glutathione (GSSG) produced upon reduction of hydrogen peroxide by GPx, further recycled to GR and NADPH. This oxidation was measured spectrophotometrically at 340 nm [12].

*References:*

1. G. Ramasatyaveni, K.B. Nimai, N.R. Dhoke, A. Das, P. Basak, Porous Polymer Scaffold for On-site Delivery of Stem Cells –Protects from Oxidative Stress and Potentiates Wound Tissue Repair, Biomater. 77 (2016) 1-13.
2. S.J Kim, S.J. Park, I.Y. Kim, T.D. Chung, H.C. Kim, S.I. Kim, Thermal characteristics of interpenetrating polymer networks composed of poly(vinyl alcohol) and poly(*N*-isopropylacrylamide), J Appl. Polymer. Sci*.* 90 (2003), 881–885. (b) C.N. Cascaval, D. Rosu, C. Ciobanu, Thermal degradation of semi-interpenetrating polymer networks based on polyurethane and epoxy maleate of bisphenol A, Polym. Test*.* 22 (2003) 45–49. (c) F.N. Kozak, O. Grigoryeva, Structure–thermal property relationships for polycyanurate–polyurethane linked interpenetrating polymer networks, Polym. Degrad. Stab. 76 (2002) 393-399. (d) E. Neilsen, Mechanical Properties of Polymers and Composites, Marcel Dekker Inc. New York, 1974.
3. W. Strobber, Trypan blue exclusion test of cell viability, Curr. Prot. In Immunol. 21 (2001) A.3B.1–A.3B.
4. V. Vichai, K. Kirtikara, Sulphorhodamine B colorimetric assay for cytotoxicity screening. Nat. Prot*.* 1 (2006) 1112 – 1116.
5. S. Archana, G. Ramasatyaveni, B.R. Narasimha, S. Satpati, G. Puroshottam, A. Panasa, A. Dixit, A. Das, A.K. Srivastava, Development of constrained tamoxifen mimics and their antiproliferative properties against breast cancer cells, BMC Letters 25 (2015) 680-684.
6. M.A. Shareef, D. Duscharla, G. Ramasatyaveni, N.R. Dhoke, A. Das, R. Ummanni, A. Srivastava, Investigation of podophyllotoxin esters as potential anticancer agents: synthesis, biological studies and tubulin inhibition properties, Eur J. Med. Chem. 89 (2015) 128-137.
7. A. Das, M.E.F.  Zapico, S. Cao, J. Yao, S.  Fiorucci, V. Hebbel, R. Urrutia, V.H. Shah, Disruption of an SP2/KLF6 repression complex by SHP is required for farnesoid X receptor-induced endothelial cell migration, J. Biol. Chem**.** 281 (2006) 39105-39113.
8. K-H.W. Lau, V. Kothari, A. Das, X. Zhang, D. J. Baylink, Cellular and molecular mechanisms of accelerated fracture healing by COX2 gene therapy: studies in a mouse model of multiple fractures, Bone 53 (2013) 369-381.
9. G.N. Adams, G.A. LaRusch, E. Stavrou, Y. Zhou, M. T. Nieman, G.H. Jacobs, Y. Cui, Y. Lu, M.K. Jain, F. Mahdi, Z. Shariat-Madar, Y. Okada, L.G. D’Alecy, A.H. Schmaier, Murine prolylcarboxypeptidase depletion induces vascular dysfunction with hypertension and faster arterial thrombosis, Blood, 117 (2011) 3929-3937.
10. H. Aebi, Catalase *in vitro*. *Methods.* Enzymol. 101 (1984) 121–126.
11. N.A. Ismail, S. H. Okasha, A. Dhawan, A.O. Abdel-Rahman, O.G. Shaker, N.A. Sadik, Antioxidant enzyme activities in hepatic tissue from children with chronic cholestatic liver disease, Saudi J. Gastroenterol. 16 (2010) 90–94.
12. N.A. Ismail, S.H. Okasha, A. Dhawan, A.O. Abdel-Rahman, O.G. Shaker, N.A. Hamid, Glutathione peroxidase, superoxide dismutase and catalase activities in children with chronic hepatitis, Adv. In Biosci. & Biotech. 3 (2012) 972-977.
